# Current understanding on the role of CCT3 in cancer research

**DOI:** 10.3389/fonc.2022.961733

**Published:** 2022-09-15

**Authors:** Wenlou Liu, Yu Lu, Xiang Yan, Quansheng Lu, Yujin Sun, Xiao Wan, Yizhi Li, Jiaqin Zhao, Yuchen Li, Guan Jiang

**Affiliations:** ^1^ Department of Oncology, Affiliated Hospital of Xuzhou Medical University, Xuzhou, China; ^2^ Department of Dermatology, Affiliated Hospital of Xuzhou Medical University, Xuzhou, China; ^3^ Department of Dermatology, The People’s Hospital of Jiawang District of Xuzhou, Xuzhou, China

**Keywords:** CCT3, TCP1, cancer, biomarker, relationship

## Abstract

Chaperonin containing TCP1 Subunit 3 (CCT3) is an important member of the chaperone protein family, providing a favorable environment for the correct folding of proteins in cell division, proliferation, and apoptosis pathways, which is involved in a variety of biological processes as well as the development and invasion of many malignant tumors. Many malignancies have been extensively examined with CCT3. It is presently used as a possible target for the treatment of many malignancies since it is not only a novel biomarker for the screening and diagnosis of different tumors, but it is also closely associated with tumor progression, prognosis, and survival. Recent studies have shown that the expression of CCT3 is up-regulated in some tumors, such as liver cancer, breast cancer, colon cancer, acute myeloid leukemia, etc. In this paper, we review the role of CCT3 in various tumors.

## Introduction

Chaperonin containing TCP1 (CCT) comprises a family of eukaryotic chaperones that have a cylindrical structure composed of two rings stacked opposite each other. Each loop consists of eight homologous but distinct subunits (CCT1-8). The eight subunits of CCT are structurally similar, consisting of a conserved equatorial domain and an actively variable apical domain (1). All these chaperones, whose equatorial domains are structurally and functionally identical, recognize different motifs in the substrate protein. Cell survival depends on CCT, which interacts with roughly 5-10% of the proteome (2). It has been proved that CCT plays an important role in the folding of many proteins involved in cancer, including Von Hippel-Lindau (3, 4) and p53 (5); proto-oncoprotein signal transduction proteins (6); and cell cycle regulatory proteins (7, 8). CCT chaperones regulate the folding of signal transduction (STAT3). In many types of cancer, STAT3 acts as a mucoprotein by delivering hormone-peptide signals to the nucleus. STAT3 has been shown *in vitro* and *in vivo* to be a substrate for the chaperone protein CCT, which contributes to its biosynthesis and activity.

CCT3 is the major subunit of the chaperone CCT complex, whose gene is located on chromosome 1. CCT3 has a molecular weight of approximately 60 kDas, and specific functions during protein folding and refolding. The CCT3 chaperone protein shares significant sequence similarities with other members of the CCT family, as well as conserved domains with other distant chaperone proteins (9, 10). The chaperone proteins play a crucial role in protein homeostasis and proteomic stability. Molecular chaperones cooperate to correctly guide protein folding pathways, intracellular localization, and proteolytic translation (11). The major functional partners of a chaperone molecule depend on their ability to transiently bind a nascent hydrophobic region or a stress-denatured polypeptide and prevent misfolding. In addition to their key role in proper folding to avoid protein mismatches, chaperones also function as targeting proteins for degradation (12). For aggregated proteins that cannot be unfolded, chaperone-mediated autophagy or a helper selective autophagy pathway is necessary to remove the damaged proteins (13, 14). Protein homeostasis is critical to normal cellular function. Thus, disrupted protein homeostasis is the basis of various diseases, such as cancer. Inevitably, molecular chaperone pathways have been implicated in the development of cancer (15, 16). Studies have shown that CCT3 might regulate insulin-like growth factor-1 (IGF-1) signaling, actin cytoskeletal signaling, and phosphatase and tensin homolog (PTEN) signaling pathways. CCT3 might also play a role in regulating microtubule structure and function (by capturing centromeres), which would affect the sensitivity of cells to these microtubule targets. CCT3 also has an important role in the tumorigenesis of epithelial cells and the growth and survival of cancer cells (17).

## CCT3 related research in tumors

Cell survival depends on the family of eukaryotic chaperones. Available information on the dysregulation of chaperone proteins reveals that CCT and its subunits are essential for the emergence of various cancers, including breast cancer (18), acute myeloid leukemia (19), hepatocellular carcinoma (20), cholangiocarcinoma (21), and colon cancer (22). **Table 1** shows the brief history of cct3 in tumorigenesis and development.

**Table 1:** A brief history of CCT3 development.

Time	Related Research and Achievements
1997	Research: Exploration of the CCT subunit. Achievement: CCT consists of 8 subunits and arrangement between subunits (1).
2001	Research: Colon cancer Achievement: As a useful tumor marker (22).
2003	Research: The non-malignant liver tissues. Achievement: CCT3 may represent targets in the progression of hepatocellular carcinoma (23).
2005	Research: Ovarian cancer Achievement: CCT3 is an effective marker for ovarian cancer (24).
2009	Research: Colon cancer Achievement: Discovery human advanced colon cancer (25).
2013	Research: Cholangiocarcinoma Achievement: CCT3 is a potential biomarker for cholangiocarcinoma (CCA) (26).
2015	Research: Breast cancer Achievement: CT3 is critical for the survival of breast cancer cells (18).
	Research: Osteosarcoma Achievement: CCT3 is candidate driver genes of importance in OS tumorigenesis (27).
	Research: Hepatocellular carcinoma Achievement: CCT3 supports proper mitotic progression and cell proliferation in hepatocellular carcinoma cells (28).
	Research: hepatocellular carcinoma Achievement: CCT3 predicts poor prognosis in hepatocellular carcinoma (29).
2016	Research: Acute myeloid leukemia Achievement: CCT modulates the activity of leukemogenic fusion oncoprotein (19).
	Research: Esophageal carcinoma Achievement: CCT3 plays an important role in the development of EC (30).
2017	Research: Gastric cancer Achievement: CCT3 is vital for gastric cancer growth (17).
2018	Research: Papillary thyroid carcinoma Achievement: CCT3 presents as a potential molecular marker of PTC (21).
	Research: Glioblastoma Achievement: CCT3 expression was significantly elevated in glioblastoma (31).
2019	Research: Tumor-repopulating cells (TRCs) Achievement: CCT3 inhibits TRCs-induced tumor formation (32).
	Research: Liver cancer Achievement: CCT3 might represent a promising biomarker for liver cancer (33).
	Research: Colorectal cancer Achievement: circ-CCT3 Enhance Colorectal Cancer Metastasis (34).
	Research: Multiple myeloma Achievement: high expression of CCT3 may serve as an indicator in diagnosis and prognosis of MM patients (35).
2020	Research: Breast cancer Achievement: Suppression of CCT3 inhibits the proliferation and migration in breast cancer cells (36).
	Research: Head and neck squamous cancer (HNSC) Achievement: CCT3 is a biomarker for improving HNSC survival and prognosis (37).
2021	Research: Hepatocellular carcinoma Achievement: CCT3-LINC00326 axis regulates hepatocarcinogenic lipid metabolism (38).
	Research: Breast and prostate cancers Achievement: CCT3 suppression prompts apoptotic machinery through oxidative stress and energy deprivation in breast and prostate cancers (39).
	Research: Lung adenocarcinoma (LUAD) Achievement: CCT3 promote cisplatin resistance of lung adenocarcinoma (LUAD) cells through the JAK2/STAT3 pathway (40).
	Research: Cervical cancer Achievement: Upregulation of CCT3 promotes cervical cancer progression through FN1 (41).

### Liver cancer

**Figure 1:**
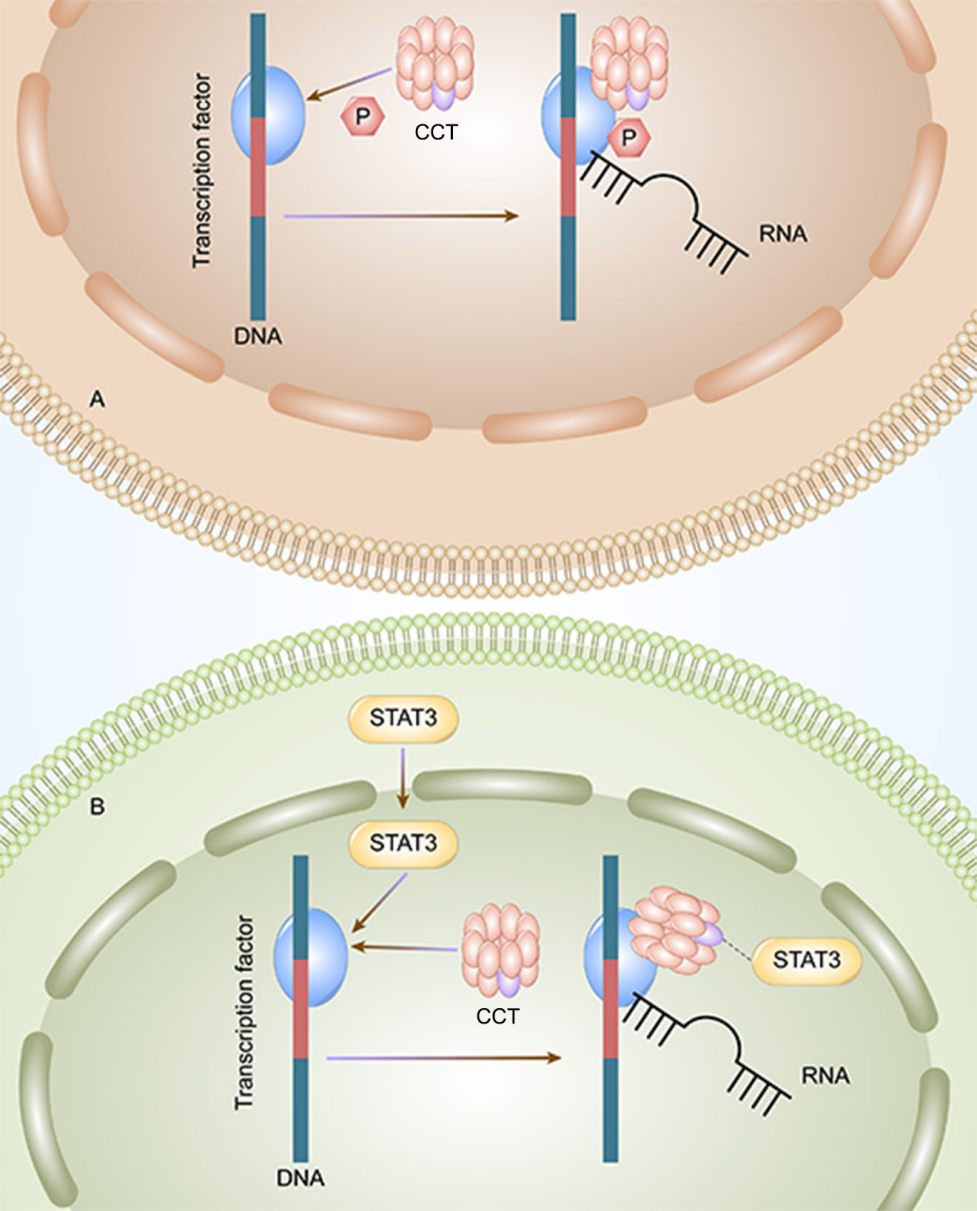
CCT3 affects the progression of liver cancer by two methods. **A**: CCT can initiate the transcription by phosphorylating the transcription factors, and the HCC tumor cells can proliferate. **B**: CCT activates STAT3 and initiates the transcription and proliferation of HCC tumor cells.

CCT3 plays an important role in the tumorigenesis and progression of HCC. The mRNA and protein expression of CCT3 in hepatocellular carcinoma (HCC) tissues are higher than those in non-HCC tissues (33). Wong et al. founded that the expression level of CCT3 in tumors was significantly higher than that of matched adjacent non-malignant liver tissues in 10 cases of HCC with amplicon 1q21-q22 (P≦0.04) (23). The PPI of CCT3-related genes is enriched in KEGG signaling pathways, indicating that CCT3 may regulate HCC by targeting related sites and gene enrichment pathways. According to the Kyoto Encyclopedia of Genes and Genomes pathway analysis, CCT3 can influence HCC occurrence and development through the cell cycle and DNA replication pathways. Through the phosphorylation of transcription factors (**Figure 1A**) and STAT3, which enter the nucleus of liver cancer cells (**Figure 1B**), CCT3 has an impact on the development of liver cancer in HCC. CCT3 is a target affecting STAT3 activation and plays a critical role in the translocation of (p)STAT3 and STAT3 from the cytoplasm into the nucleus. HCC progression is partly affected by knockdown of CCT3, which may negatively regulate activation of the IL6/STAT3 signaling pathway and affect the progression of HCC partly by having an impact on the transport of (p)STAT3/STAT3 into the nuclei of HCC cells (42). By maintaining proper chromosome alignment and segregation (29), CCT3 can play a part in regulating microtubule structure and function (capturing the attachment site). By preventing the ubiquitination of YAP and TFCP2 brought on by poly (RC) binding protein 2 (PCBP2) in a beta-transducin repeat containing E3 ubiquitin protein ligase (trcp)-independent way, CCT3 extended their half-lives (28). YAP and TFCP2 are recognized as upstream triggers to increase the protein stability of YAP and TFCP2 in liver cancer cells. Bioinformatics prediction and luciferase reporter assays validated that circ-CCT3 could promote HCC progression through the mir-1287-5p/TEA domain transcription factor 1 (TEAD1) axis (43). Circ-CCT3 acted as a sponge for mir-1287-5p to enhance TEA domain transcription factor 1 (TEAD1) expression, which subsequently contributed to the activation of PTCH1 and LOX and consequently promotes tumorigenesis and progression. In addition, CCT3 can be associated with hepatocyte lipid metabolism in cells and *in vivo via* the long-stranded non-coding RNA LINC00326. Perturbation of the CCT3-LINC00326 regulatory network results in decreased intracellular lipid accumulation and increased lipid degradation, as well as diminished tumor growth *in vivo* (38).

Analysis of some recent cancer-driven genes identified CCT3 as a novel biomarker for liver cancer screening and diagnosis (44). In particular, for AFP-negative and small HCC patients, the CCT3 protein level showed good correlation with HCC etiology, tumor size, TNM stage, and Child-Pugh classification. The progression of malignancy is positively linked with the degree of CCT3 expression (29, 42). CCT3 overexpression is associated with poorer clinical outcomes and aggressive clinicopathological features, suggesting a poor prognosis for patients with HCC (33). In addition, CCT3 contributed to the invasion capacity of cells. This showed that CCT3 expression may be associated with metastasis in HCC. In conclusion, CCT3 is involved in the carcinogenesis and progression of HCC and serves as a potential therapeutic target and biomarker in hepatocellular carcinoma (28, 33).

### Breast cancer

CCT3 was significantly upregulated in a large proportion of human breast cancer tissues, and its overexpression was also significantly correlated with breast cancer clinical characteristics, including the clinical stage and the TNM classification. Both the mRNA and the protein levels of CCT3 are potential diagnostic biomarkers and therapeutic targets for breast cancer (45). Xu et al. found that CCT3 regulates breast cancer tumorigenesis by promoting cell proliferation and cell cycle progression. The knockdown of CCT3 inhibited proliferation, metastasis, and apoptosis of breast cancer cells, and the mechanism may be related to the regulation of the cell cycle, apoptosis, and multiple signal transduction pathways (36). They confirmed that the knockdown of CCT3 can induce apoptosis in breast cancer with the annexin method in this study. Perhaps the mechanism is related to CDC20 and p53. They are substrates of CCT. CDC20 is known to modulate key anti-apoptotic proteins Mcl-1 and Bim (46, 47), and p53 mediates cell apoptosis by activating the mitochondrial pathway and death receptor-induced apoptosis pathway (48). Secondly, down regulation of CCT3 significantly inhibited NF-κB activity and reduced the proliferation and metastatic capacity of breast cancer cells (30, 36). Qu et al. further explored the mechanism of action of CCT3 in breast cancer was performed through gain-and loss-of-function studies and found that CCT3 can directly bind mir-223 through the ceRNA network between mir-223 and β-catenin, thus affecting the activation of the Wnt/β-catenin signaling pathway, attenuating the regulation of mir-223 in the Wnt/β-catenin pathway and promoting breast cancer cell proliferation and tumorigenicity (45). In this study, they found that CCT3 can promote β-catenin nuclear translocation, and there is a high possibility that CCT3 may recruit β-catenin into the nucleus through direct binding. Wnt/β-catenin signaling was activated when CCT3 was knocked down. The target genes downstream, such as cyclin D1 and c-myc, were then transcribed with the help of β-catenin. These Wnt/β-catenin target genes promote breast cancer cell G1/S transition and other oncogene transcriptions, maintaining the malignant proliferative ability (**Table 2**). In breast and prostate cancers, miRNA-mediated CCT3 inhibition can disrupt intracellular ROS homeostasis, leading to elevated ROS levels, altering intracellular free amino acid water and distribution for energy metabolism and promoting apoptotic mechanisms through oxidative stress and energy deprivation (39).

**Table 2:** CCT3 can affect tumor progression through STAT3, cdc20, p53, NF-κB, the wnt pathway, the VEGF pathway.

Signaling pathway or transcription factor	Progression	Impact on cancer
JAK/STAT3	STAT3 is an important downstream signaling molecule of numerous growth factors and cytokines, and participates in various biological processes, such as cell proliferation, differentiation, and survival. STAT3 can be activated by nonreceptor tyrosine kinases such as Janus kinases (JAKs) in a tyrosine phosphorylation dependent-manner. CCT3 down regulation could sensitize lung adenocarcinoma cells to cisplatin by inhibiting the Janus kinase 2/signal transducer and activator of the transcription 3 (JAK2/STAT3) pathway.	CCT3 overexpression might affect the progression of multi-ple myeloma through the JAK/STAT3 pathway. The JAK2/STAT3 pathway has been considered a promising target for chemotherapeutic interference ascribed to its persistent activation in human carcinomas. CCT3 may be a new molecular target to overcome cisplatin resistance of LUAD patients
Cdc20,P53	Cdc20 is known to modulate key anti-apoptic proteins Mcl-1 and Bim, and p53 mediates cell apoptosis by activating mitochondrial pathway and death receptor-induced apoptotic pathway. CDC20 was frequently upregulated in many types of malignancies and remarkably suppressed by ectopic introduction of p53.	P53 inhibits tumor cell growth through the indirect regulation of CDC20 and that CDC20 might be a good potential therapeutic target for a broad spectrum of human cancer.
NF-κB	Down regulation of CCT3 significantly inhibited NF-κB activity and reduced the proliferation and metastatic capacity of breast cancer cells	Overexpression of NFκB rescued the effect of CCT3 on the proliferation and migration of breast cancer cells.
The Wnt/βpathway	Wnt/β-catenin is a key signaling pathway in cancer cell proliferation. Wnt signaling was highly activated in MDA-MB-231 and T47D cells when CCT-3 was upregulated. In contrast, knocking down of CCT-3 knocked down the Wnt signaling significantly. The protein level of p-GSK- 3β and β-catenin nuclear accumulation increased in CCT3-overexpressed MDA-MB-231 and T47D cells. CCT-3 also affected the expression levels of β-catenin downstream effectors such as cyclin D1 and c-myc.	CCT-3 may promote breast cancer tumorigenesis at least in part *via* activating the Wnt/β-catenin signaling pathway.
The VEGF pathway.	Circ-CCT3 depletion attenuates invasion and induces apoptosis of CRC cells through mir-613/WNT3 or VEGFA. Thus, circ-CCT3 can enhance colorectal 173 cancer metastasis by regulating VEGFA.	Circ-CCT3 plays an oncogenic role in CRC metastasis through mir-613/VEGFA and Wnt signaling.

### Lung cancer

Investigations according to the GEPIA (Gene Expression Profiling Interactive Analysis) web portal demonstrated that CCT3 expression levels were significantly upregulated in both lung adenocarcinoma (LUAD) and lung squamous cell carcinoma (LUSC) tissues. Furthermore, it was concluded from the Cancer Genome Atlas (TCGA) and Genotype-Tissue Expression (GTEx) databases that a high expression level of CCT3 was associated with the poor prognosis of LUAD patients, albeit not in LUSC patients. Therefore, Xu et al. speculated that CCT3 may be closely involved in the tumorigenesis and progression of non-small cell lung cancer (NSCLC), and it might have a more prominent role in LUAD (40). Shi et al. found through further studies revealed that silencing of CCT3 resulted in the inhibition of Yes-associated protein 1 (YAP1) in NSCLC cells, decreasing the expression of YAP1 target genes and producing antitumor effects in NSCLC. In NSCLC cells, activation of the YAP1 by forced expression of constitutively active YAP1 mutants reversed the antitumor effects induced by CCT3 inhibition. This study unveils a possible role for the CCT3/YAP1 axis in NSCLC and suggests CCT3 as a candidate anticancer target (49). The increased expression of CCT3 in lung adenocarcinoma may lead to uncontrolled cell proliferation by promoting the expression of cyclin B1/CDK1 and thus accelerating cell cycle progression (40). Under cisplatin treatment, cell cycle protein B1 and CDK1 protein levels in lung adenocarcinoma cells were significantly reduced after CCT3 knockdown. The downregulation of CCT3 significantly inhibited the proliferation, invasion, and migration of lung adenocarcinoma cells, resulting in increased apoptosis and enhanced expression of the apoptosis marker cleaved caspase-3 (34) and leading to significant G2/M cell cycle arrest and apoptosis in lung adenocarcinoma cells, significantly reducing the tumorigenicity of cisplatin-treated lung adenocarcinoma cells. In addition, CCT3 downregulation could sensitize lung adenocarcinoma cells to cisplatin by inhibiting the Janus kinase 2/signal transducer and activator of the transcription 3 (JAK2/STAT3) pathway. It is suggested that CCT3 may be a new molecular target to overcome cisplatin resistance in LUAD patients (40) (**Table 2**).

### Cervical cancer

Dou et al. examined the effect of CCT3 on the proliferation and migration of cervical squamous cell carcinoma and endocervical adenocarcinoma (CESC) *in vitro* through various experiments, including proliferation, Transwell, and flow cytometric assays. The results showed that CCT3 expression was significantly upregulated in CESC. *In vitro*, silencing CCT3 inhibited the proliferation, migration, and invasion of CESC cells. Downregulation of the CCT3 gene promoted apoptosis and cell cycle arrest in CESC cells and inhibited the expression of fibronectin 1 (FN1) protein. Furthermore, rescue assays demonstrated that CCT3 could promote the proliferation and migration process of CESC through FN1 (50). Saioa Mendaza et al. experimentally found that mir-877-3p promotes Squamous Cell Carcinoma of the Cervix (SCCC) cell migration and invasion by regulating cytoskeletal protein folding, mainly through the CCT complex, and that anti-mir-877-3p increased the expression of CCT3 in SCCC, leading to abnormal folding of actin and microtubulin, thereby impairing cell migration and invasion ability. Overall, CCT3 may be considered a new and promising biomarker that is closely related to the progression, prognosis, and survival of CESC and may become a therapeutic target for CESC (26).

### Multiple myeloma

Qian et al. study analyzed 2220 multiple myeloma patients (2380 samples) using 10 independent GEO datasets with different bioinformatics analysis methods and found that CCT3 was significantly overexpressed in multiple myeloma patients and that CCT3 overexpression was a poor predictor of survival, and its functional intensity was positively correlated with disease progression. CCT3 may play a supporting role in myeloid diagnosis. KEGG pathway analysis indicated that the CCT3 targeted genes were involved in the JAK-STAT3 signaling pathway, the Hippo signaling pathway, the WNT signaling pathway, and two pathways centralizing in leukemia-related terms (namely acute myeloid leukemia and chronic myeloid leukemia) (41, 51, 52). GSEA of gene sets differentially regulated in the CCT3 high and CCT3 low groups revealed that leukocyte migration, regulation of leukocyte migration, IL6/JAK/STAT3 signaling, and regulation of STAT cascade gene sets were significantly upregulated in the CCT3 high group. These results suggest that high CCT3 expression is associated with leukemia and the JAK-STAT3 signaling pathway, and that CCT3 expression may promote multiple myeloma progression by regulating MYC mainly through the JAK-STAT3 signaling pathway. These findings suggest that high CCT3 expression may serve as an indicator for the diagnosis and prognosis of multiple myeloma patients and a potential target for the future treatment of multiple myeloma (53).

### Papillary thyroid carcinoma

Susannah Hallal et al. used TCGA data to interrogate gene expression levels and DNA copy numbers *in silico* for all eight subunits. Glioblastoma tissue had significantly higher levels of CCT2, CCT3, CCT5, CCT6A, and CCT7 gene expression relative to healthy brain tissue (52, 54). Human papillary thyroid cancer (PTC) tissues had significantly greater levels of CCT3 expression than did healthy paraneoplastic tissues (21). CCT3 is essential for the survival and growth of PTC cells, and the lentivirus-mediated knockdown of the CCT3 gene significantly reduced the proliferation and cell cycle progression of K1 cells and induced apoptosis in K1 cells. In addition, enhanced G2/M cycle blockade could also lead to increased apoptosis after CCT3 knockdown. The current findings suggest that CCT3 is a significant oncogene in PTC, and it was found that an increase in CCT3 expression was associated with the tumor area of PTC. In conclusion, CCT3 may be a promising potential candidate gene for molecular diagnosis and treatment of PTC and may serve as a predictive prognostic indicator for PTC patients and a molecular target for PTC treatment (21).

### Malignant melanoma

Protein is the actual functional molecule in cells and is responsible for almost all the biochemical activities of cells and is the basis of biological functions. CCT3 regulates the pathogenesis and metastasis of melanoma at the protein level. Roobol et al. reported that CCT3 is an important partner of the folded cytoskeletal component, and elevated CCT3 expression might impair the proper folding and assembly of complex proteins (35). CCT3 may regulate IGF-1 signaling, actin cytoskeletal signaling, and PTEN signaling pathways, which are known to function in epithelial tumorigenesis and cancer cell growth and play an important role in survival. CCT3 might play a role in regulating microtubule structure and function (capturing centromeres), which would affect the sensitivity of cells to these microtubule targets. In addition, cytoskeletal proteins (27), including actin and tubulin (31, 37), many cell cycle regulators (7), and tumor suppressors (37), are also substrates of CCT.

Numerous studies have shown that many proteins play a key role in the occurrence and metastasis of malignant melanoma (MM). Baruthio et al. detected a total of more than 100 proteins in the detergent-resistant membrane of melanoma cell lines, and melanoma cells are particularly resistant to detergents. Membrane protein structure is related to the degree of malignancy, suggesting that some membrane proteins might play a role in the progression of melanoma (55). Sinha et al. used two-dimensional electrophoresis and proteomics methods to analyze the protein expression profiles of drug-resistant melanoma cells and found that voltage-dependent anion channel proteins were overexpressed. Molecular chaperones were also overexpressed in resistant melanoma cells, including heat shock proteins (32). BRaf proto-oncogene, serine/threonine kinase (BRAF) is the most frequently mutated serine-threonine protein kinase involved in the pathogenesis of melanoma and is mutated in most melanoma and melanocyte lesions. However, BRAF mutations mainly occur in the progression of melanoma and rarely occur in the advanced stages, suggesting that this pathway plays a role in the initiation and progression of melanoma (56). The dephosphorylated protein cuts the filaments near the serosa to cause actin to polymerize to create new spikes to promote melanoma cell invasion.

**Figure 2:**
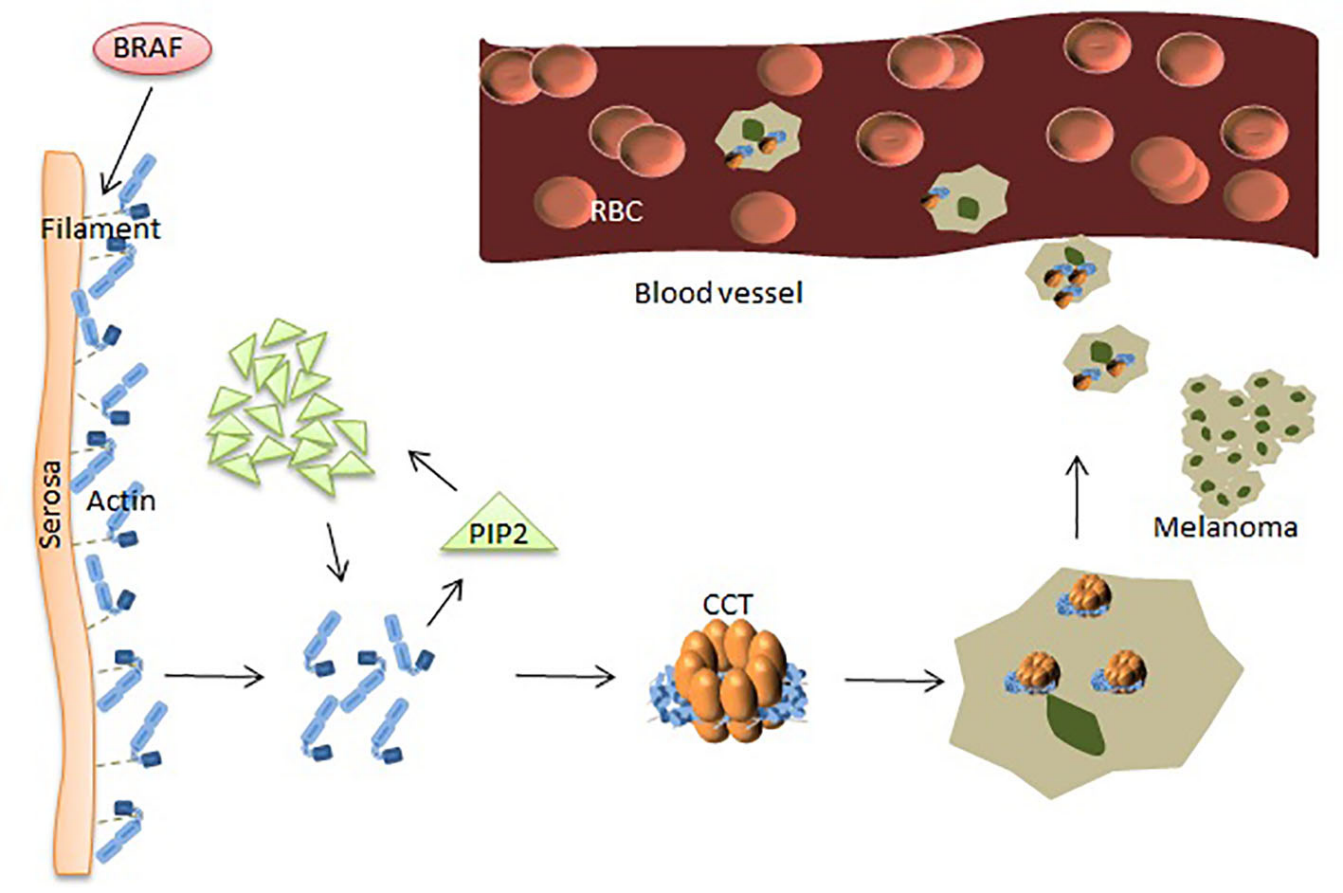
CCT plays a key role in the progression of melanoma. BRAF makes dephosphorylated protein cut the filaments near the serosa, causing polymerization of actin monomers. The increased amount of actin acts on PIP2, and the clustered PIP2s, in turn, increase actin monomers. Polymerized actin binds between the two rings of CCT and provides the driving force for movements leading to MM cells’ metastasis.

In other settings, increased levels of plasma membrane intrinsic protein 2 (PIP2) can be achieved by binding and replacing actin filament capping proteins, such as gelsolin, at the ends of actin filaments (high levels of gelsolin reduce melanoma cell metastasis), thereby promoting actin polymerization. The mechanism of actin polymerization is dependent on the availability of intracellular actin monomers, which are associated with melanoma metastasis (24) (**Figure 2**). The structure of actin is V-shaped, and the two arms are composed of two large and one small domain, and the gap between them is visible with nucleotide (ATP) adhesion. CCT binds to ATP and has weak ATPase activity. After actin enters the CCT side cavity, it binds tightly to the apical domain of CCT through the arm of the large domain. The driving force for cell body movement comes from the interaction of actin and myoglobin, which is mainly regulated by phosphorylation of non-muscle cell types. Many phosphorylation pathways that affect regulatory myosin subunits are up-regulated in metastatic melanoma cells. Rhoc increases actin-myosin contractility by binding to ROCK1 and ROCK2, and high expression of Rhoc is associated with melanoma metastasis. Inhibition of rhoc reduces the invasiveness of melanoma cells (57). Experiments have shown that the microtubule network acts on the adhesion point to separate it, which is beneficial to the protein transfer induced by dynein. Homomorphic tubulin is homomorphic. It enters the inner cavity of the CCT side loop to form a CCT-tubulin complex. When tubulin binds to the CCT side loop, it stops the information exchange of the loops, preventing the other loop from binding to other substrates. Some proteins are currently used for the diagnosis of MM. However, the diagnosis of melanoma by using a single protein index remains insufficient.

### Other tumors

At present, there are many researches on CCT3 in liver cancer, breast cancer, lung cancer, and so on. There has also been some research into other tumors.

#### Gastric cancer

CCT3 was found to be important in the growth and survival of gastric cancer. Li et al. used immunohistochemistry to show that CCT3 expression levels were found to be higher in surgical specimens from 26 gastric cancer patients than in non-cancerous epithelial tissue adjacent to cancer. RNA interference was used to knock down the expression of CCT3 in gastric cancer cell lines. Cellular knockdown of CCT3 inhibited proliferation and colony formation, reduced cell viability, and promoted apoptosis of gastric cancer cells *in vitro*. Gene expression analysis showed that CCT3 knockdown was associated with down-regulation of mitogen-activated protein kinase 7, cell division cycle 42, cyclin D3 and up-regulation of cyclin-dependent kinase 2 and 6 (17).

#### Colorectal cancer

Li et al. used reverse transcription-quantitative PCR and western blot to detect the mRNA and protein levels of each gene and found that circ-CCT3 was highly expressed in human clinical CRC tumors. Low levels of circ-CCT3 are strongly associated with higher survival and tumor metastasis in CRC patients. Circ-CCT3 plays an oncogenic role in CRC metastasis through mir-613/VEGFA and Wnt signaling. Mechanistically, circ-CCT3 directly interacts with mir-613, then regulates VEGFA and WNT3 gene expression. Phenotypically, circ-CCT3 depletion attenuates invasion and induces apoptosis of CRC cells through mir-613/WNT3 or VEGFA. Thus, circ-CCT3 can enhance colorectal cancer metastasis by regulating VEGFA and WNT3 signaling through sponge uptake of mir-613 (25, 34) (**Table 2**).

#### Esophageal cancer

Su et al. quantitative real-time polymerase chain reaction (qPCR) was used to verify the expression level of differentially expressed genes (DEGs) in esophageal carcinoma (EC) and identified significant differentially expressed genes (DEGs) of CCT3 that play a key role in the tumor development of esophageal cancer (EC). CCT3 can also be considered as a potential candidate biomarker for therapeutic targets of esophageal cancer (30). A proteomics-based study by Shi et al. showed that CCT3 is a potential biomarker for cholangiocarcinoma (CCA) and has the potential to be used as a new tumor marker for the early detection of CCA (58).

#### Osteosarcoma

Xiong et al. concluded from a comprehensive analysis of osteosarcoma (OS) gene expression and genomic aberration data that CCT3 is an important pivotal protein in the development of osteosarcoma and plays an important role in the progression of osteosarcoma. Furthermore, CCT3 is an important specific driver gene in osteosarcoma. It can be an excellent candidate biomarker for OS diagnosis and may be used to improve the diagnostic markers for neonatal osteosarcoma (59).

#### Head and neck squamous cell carcinoma

The GEO, Oncomine, and ALCAN databases were used to examine CCT3 expression in head and neck squamous cell carcinoma (HNSCC). The results showed that CCT3 expression was significantly up-regulated in HNSCC at both mRNA and protein levels. Up-regulated CCT3 expression was associated with various clinicopathological parameters. Gene set enrichment analysis (GSEA)analysis indicated that high expression of CCT3 was closely correlated with tumor-related signaling pathway mTOR pathway (MTORC1/PI3K AKT mTOR) and HNSCC cell survival. In addition, overexpression of CCT3 was associated with unfolded protein response, DNA repair, and the p53 pathway, which may contribute to the progress of HNSCC. Cellular knockdown of CCT3 significantly inhibited cell growth and invasion of HNSCC cell lines. Thus, CCT3 could be a prognostic marker and potential therapeutic target in HNSCC (60, 61).

#### MCKD1

Wolf et al. collected clinical data and blood samples from 257 individuals (including 75 affected individuals) from 26 different relatives. Mutation analysis was performed on 37 genes (374 exons) in 23 patients with MCKD1 in a defined critical region. In addition, for nine relatives, RT-PCR analysis of the sequenced genes was performed to screen for mutations that activate hidden splice sites. Mutational analysis of all 37 positional candidate genes revealed sequence variation in CCT3, which was isolated from each affected relative and was not found in 96 healthy individuals, suggesting that CCT3 is important to MCKD1 (62).

#### Ovarian cancer

Peters et al. experimentally identified several potential novel biomarkers for ovarian cancers, including CCT3. Taken together, these data identified no elevated expression of genes in primary ovarian cancer but confirmed a valid existing marker, which is CCT3 (63).

### Study of tumor-repopulating cells

Tumor-repopulating cells (TRCs) are cancer stem cell (CSC)-like cells with highly tumorigenic and self-renewing abilities. Huang et al. identified CCT3 as a potential new stem cell-associated gene by integrating a network of membrane proteins, the WNT pathway, and cancer-related genes. Not only is it a key molecule in the selection of tumor-repopulating cells (TRCs), but it is also important for mechanotransduction and influences the selection process of TRCs. The number of TRC colonies was dramatically decreased by silencing CCT3, and TRC growth and selection were also slowed. Additional research revealed that CCT3 increased stemness and cell proliferation *in vitro*, while its inhibition prevented the development of tumors brought on by TRCs (64).

## Conclusion and perspectives

In this review, we first briefly introduce the structure and function of CCT, and then focus on the role of CCT3 in a variety of tumors. We discuss the multiple regulatory mechanisms involved in CCT3 in eukaryotes. For example, overexpression of CCT3 promotes the proliferation and differentiation of cancer cells and induces apoptosis, thereby inhibiting cell proliferation. CCT3 has been shown to mediate the folding of many proteins involved in tumorigenesis. Great progress has been made in elucidating the role of CCT3 in tumorigenesis through STAT3, Wnt, PI3K-AKT and other related signaling pathways and its expression mechanism in diseases. Although the experiments of tumor therapy and prognosis targeting CCT3 have been carried out widely and achieved some results, further work is needed to study the role of CCT3 as a tumor marker and drug-targeted therapy in tumors. It can be used to develop strategies to identify new cancer therapies. Therefore, it is necessary to develop new methods and further experimental studies to improve our understanding of the role of CCT3 in tumor development and progression.

## Author contributions

GJ and WLL provided direction and guidance throughout the preparation of this manuscript. WLL drafted the manuscript. YL, XY and QSL designed the tables and imagines. YJS ,YZL, YCL and JQZ collected and prepared the related literature. GJ and WLL reviewed and made significant revisions to the manuscript. All authors contributed to the article and approved the submitted version article and approved the submitted version.

## Conflict of interest

The authors declare that the research was conducted in the absence of any commercial or financial relationships that could be construed as a potential conflict of interest.

## Publisher’s note

All claims expressed in this article are solely those of the authors and do not necessarily represent those of their affiliated organizations, or those of the publisher, the editors and the reviewers. Any product that may be evaluated in this article, or claim that may be made by its manufacturer, is not guaranteed or endorsed by the publisher.

## Abbreviations

CCT: chaperonin containing t-complex; TCP1: T-complex protein 1; HCC: hepatocellular carcinoma.
